# Evaluation of Methods Employed in Establishing Preclinical Similarity of Adalimumab Biosimilars

**DOI:** 10.1155/adpp/8816591

**Published:** 2025-06-09

**Authors:** Ramya Nair, Naveen Krishnan, Vasudev Shenoy, Raviraja N. Seetharam

**Affiliations:** ^1^Manipal Centre for Biotherapeutics Research, Manipal Academy of Higher Education, Manipal 576104, Karnataka, India; ^2^BA/BE Services, Navitas Life Sciences, Manipal 576104, Karnataka, India

**Keywords:** analytical, biologics, biosimilar, humira, preclinical

## Abstract

Adalimumab, marketed as Humira, is a fully humanized monoclonal antibody that blocks the activity of tumor necrosis factor-alpha and is used in treating several autoimmune disorders. As one of the top-grossing pharmaceuticals, its global sales surpassed $20 billion in 2023, leading to significant biosimilar development, with 10 products available by 2025. This review analyses published preclinical studies to assess the evaluation methods employed to establish biosimilarity between Humira and four key biosimilars: ABP501 (Amjevita), FKB327 (Hulio), MSB11022 (Idacio), and SB5 (Imraldi). Our comparative analysis reveals that primary structure, glycosylation profiles, Fc receptor binding affinity, and TNF-alpha neutralization potency are critical quality attributes essential for establishing biosimilarity. Notably, while all four biosimilars demonstrated comparable functional properties to the reference product, variations in glycosylation patterns presented distinct regulatory challenges. This review is a valuable resource for biopharmaceutical scientists engaged in biosimilar development, ultimately supporting advancing more accessible and affordable treatment options while ensuring adherence to stringent efficacy, safety, and quality standards of adalimumab biosimilars.

## 1. Introduction

Tumor necrosis factor *α* (TNF *α*) is a cytokine secreted by macrophages that regulates several biological functions, including cell growth, differentiation, and apoptosis. They are associated with the development of autoimmune disorders, psoriasis, insulin resistance, and cancer development [1]. The role of TNF *α* was first reported by William Colet in 1891, after the remission of disseminated sarcoma after *Streptococcus* infection [2], and was later cloned in 1984 [3]. Even though TNF *α* is widely acknowledged and approved, its clinical applications remain restricted due to its potential toxicity including systemic shock and inflammatory responses [4].

The effects of TNF *α* are mediated by TNF receptors I and II, which are present in all cells except erythrocytes [5]. Binding of TNF *α* to these receptors activates nuclear factor-κβ, caspases, and protein kinases, including c-Jun N-terminal kinase and MAP kinase [5]. This stimulates the secretion of cytokines, including inflammation and immune responses, and triggers apoptosis. The biological impacts of TNF *α* include activating various immune cells (macrophages, T cells, and B cells), producing proinflammatory cytokines (IL-1 and IL-6) and chemokines (IL-8 and RANTES) [6]. Soluble TNF *α* and its precursor, transmembrane TNF *α*, have different modes of action [6]. Transmembrane TNF *α* functions through direct cell-to-cell contact, while soluble TNF *α* works at distant sites from its production cells [7].

**Figure 1:**
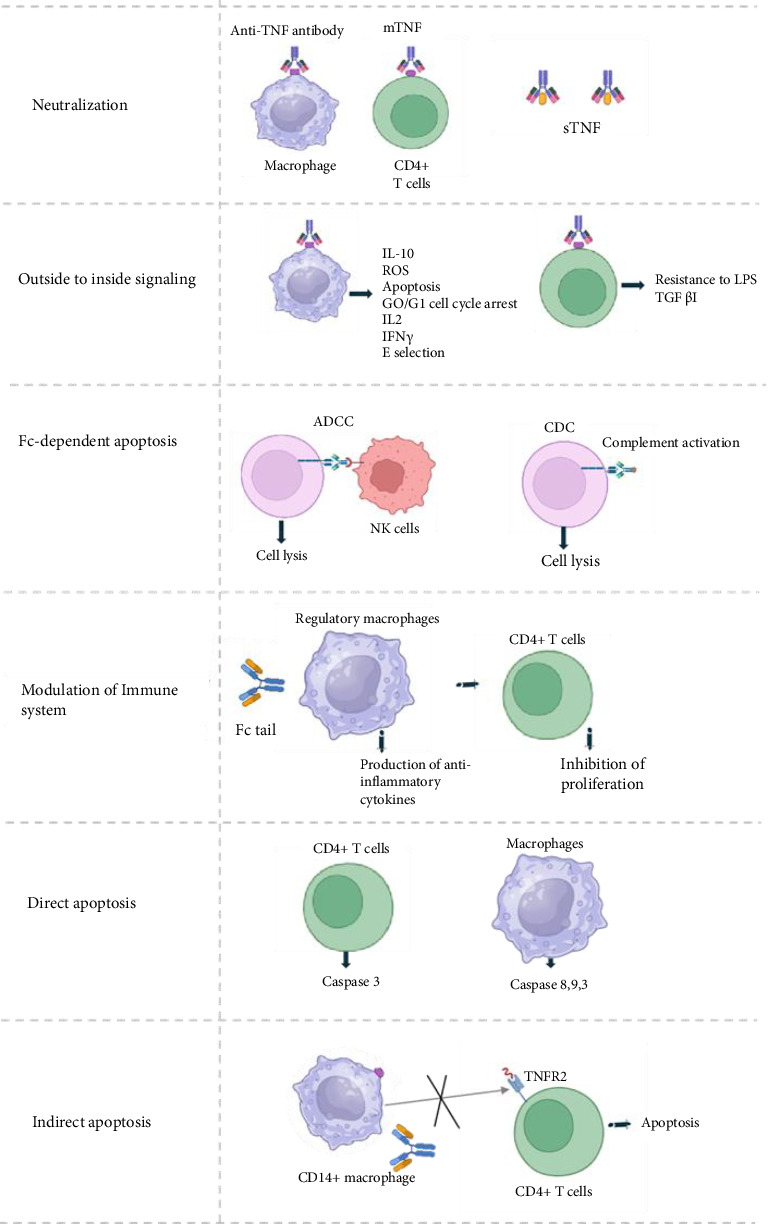
Mechanism of action of TNF*α* inhibitors.

TNF *α* inhibitors are used as immunotherapy agents for treating autoimmune conditions, and they function by preventing the binding of TNF *α* to the receptors through five proposed mechanisms [8] (Figure 1). In the first neutralization mechanism, the binding of soluble TNF *α* with T-cells and macrophages is blocked. In the second mechanism, the outside-to-inside signaling, binding of transmembrane TNF *α* is blocked. The inhibitors also can target the Fc-dependent apoptosis pathway, which attaches to the Fc domain to prevent the release of cytotoxic proteins such as perforins and granzymes. Monoclonal antibody inhibitors also blocks the release of TNF cytokines such as IL-13, IL-17, or IFN-γ or target the apoptosis induced by TNF signaling that occurs through complement or natural killer cells [7].

Etanercept was the first TNF *α* inhibitor, approved on August 24, 1998, for treating rheumatoid arthritis (RA) [9]. The introduction and integration of TNF *α* inhibitors into clinical practice marked a significant milestone in the therapeutic approach to autoimmune diseases. By 2008, the annual revenue from the five approved TNF *α* inhibitors (etanercept, infliximab, adalimumab, certolizumab pegol, and golimumab) exceeded 16 billion US dollars. Furthermore, in 2012, etanercept, infliximab, and adalimumab emerged as the top-selling drugs across all therapeutic categories [6]. Humira, a 2002-approved innovative TNF *α* inhibitor developed by AbbVie, has established itself as the leading TNF *α* inhibitor in the global market. Its primary indications include the treatment of RA, psoriatic arthritis, ankylosing spondylitis, Crohn's disease, ulcerative colitis, plaque psoriasis, hidradenitis suppurativa, uveitis, and juvenile idiopathic arthritis [10]. The prevalence of these conditions worldwide ranges from 0.24 to 1% for RA, 0.1% to 1% for psoriatic arthritis, and 0.3% for IBD, Crohn's, and ulcerative colitis.

Humira is the most expensive prescription medication in the United States, with annual expenditure exceeding $84,000. Since its market entry, the price has escalated by 470% [11]. Since its launch, it has consistently ranked among the top-selling biopharmaceuticals in the US and European markets, maintaining its position as the exclusive adalimumab brand for autoimmune conditions for 14 years. Due to its cost and vigorous patent protection, it was unavailable to the general public. However, following the expiration of its initial patent in 2016, due to the high demand, other manufacturers picked up momentum. They started marketing biosimilars, a cost-effective equivalent; by 2024, there will be 10 approved biosimilars to Humira (Table 1).

Biosimilars represent a significant step toward achieving healthcare parity, offering hope for health equality. Biotherapeutics are being incorporated into the WHO Model list of essential medicines despite being expensive and facing challenges in economically weaker nations. This move aims to expedite regulatory approval and facilitate the entry of biosimilars into the markets of low- and middle-income countries. It focuses on improving supply chains, procurement processes, and health system requirements. By encouraging the knowledge transfer, biosimilars are given priority to ensure better access and affordability.

**Figure 2:**
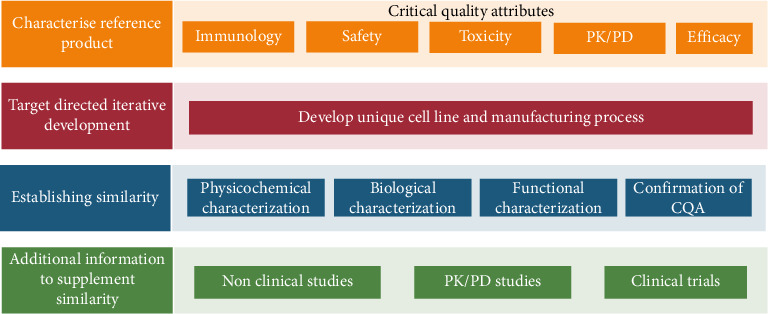
Schematic of biosimilar development.

**Figure 3:**
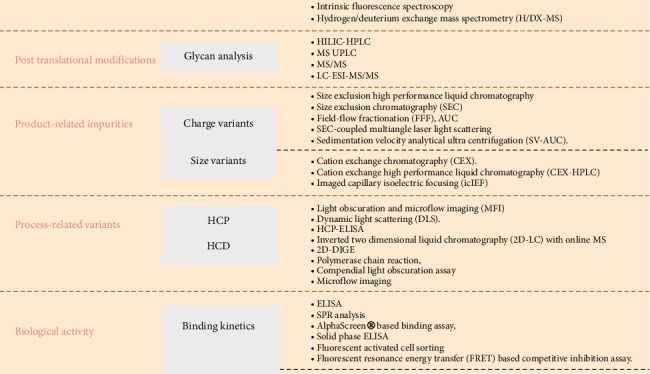
A comprehensive representation for evaluating critical quality attributes and biological activities in biosimilar assessment.

The biosimilar development faces substantial challenges, including the exclusive nature of the original products, their manufacturing processes, and the complexity of biological molecules [12]. A schematic representation of biosimilar development is given in Figure 2. Unlike small-molecule generic products, replicating identical copies of biologic products is unattainable [13]. However, to ensure quality production, regulatory guidelines require biosimilar producers to comply with the latest industry norms and regulatory demands that are constantly evolving. The European Medicines Agency (EMA) and the US Food and Drug Administration (FDA) provide detailed requirements for comprehensive similarity evaluations to prove equivalence to the original product [14, 15]. During the production of the biosimilar for Humira, a therapeutic monoclonal antibody, it is crucial to confirm the structural similarity (primary and higher-order structures [HOSs] as well as charge and size) with reference products. The functional and analytical assays conducted during the preclinical stage are essential for demonstrating this similarity and play a key role in the regulatory approval process. In addition, purity assessments, in vitro functional assays, and in vivo animal studies provide analyses comparing biosimilar's physicochemical, analytical, and functional characteristics to the reference products [16]. This review provides a comprehensive analysis of articles comparing the physicochemical, analytical, functional, and biological assays and their biological comparison to the reference product (Figure 3).

## 2. Analytical Biosimilarity Analysis

In assessing biosimilars, evaluating their structural and functional similarities to reference products is critical. Various analytical and functional characterization methods that adhere to regulatory guidelines are used during evaluation [16]. This includes the use of high-resolution, complementary analytical tools for qualitative validation. In quantitative analysis, the critical quality attributes (CQAS) are analyzed. Key quality attributes of biotherapeutic products can be categorized into several areas, including primary and HOSs, glycosylation patterns, and variants associated with the product and the manufacturing process. This approach ensures that biosimilars are adequately compared with their reference products, maintaining safety and efficacy standards [17]. The primary structure covers intact or subunit mass, amino acid sequencing, and disulfide analysis. HOS includes secondary and tertiary structures, along with stability. In glycosylation analysis, oligosaccharide patterns, glycopeptide mapping, and monosaccharide/sialic acid contents are examined. Variations in size, charges, and protein alterations due to posttranslational modifications (PTMs) leading to aggregation, fragmentation, and loss of N-terminal and C-terminal proteins are analyzed in product variants.

Process-related variants include contaminants such as host cell proteins (HCPs) and host cell DNA (HCD). When evaluating biosimilarity, the biosimilar drug substance/drug product and the innovator's structural fingerprints are compared. The evidence's strength depends on the resolution technique and sensitivity. A single method may not suffice for a multifaceted attribute, such as HOS and size variants. USFDA guidelines state that each comparative analytical platform, from biophysical tools to new technologies, has unique pros and cons [18].

### 2.1. Primary Structure Analysis: Adalimumab (Humira) Biosimilars

The primary structure analysis involves meticulously examining the fundamental protein structure by examining the precise sequence of amino acids composing the protein. In the biosimilarity assessment of monoclonal antibodies such as Humira (adalimumab), confirming the amino acid sequence is conserved is essential [19].

#### 2.1.1. Molecular Mass Analysis

The precise characterization of amino acid sequences and molecular masses can be performed using several techniques such as SDS-PAGE, size-exclusion chromatography (SEC), and matrix-assisted laser desorption/ionisation time-of-flight mass spectrometry (MALDI-TOF-MS) [18]. In SDS-PAGE, proteins are separated based on size, using sodium dodecyl sulfate to denature proteins and a polyacrylamide gel to resolve them. Conversely, SEC utilizes a porous matrix to separate molecules according to size, enabling smaller molecules to penetrate the pores and migrate faster, thus eluting first [20].

MALDI-TOF-MS uses a laser to ionize molecules, which are then separated by their mass-to-charge ratio in a time-of-flight analyzer, and it was initially used for analyzing the molecular mass of biosimilars. However, it struggled with interference from high-abundance proteins and offered a limited resolution for complex glycoproteins [21]. SEC and SDS-PAGE failed to resolve subtle mass differences due to PTMs or aggregation while distinguishing the biosimilars from reference products [22].

The advent of electrospray ionization time-of-flight mass spectrometry (ESI-TOF-MS) revolutionized molecular mass analysis by enabling high-resolution intact mass measurements with mass errors as low as ≤ 4 ppm [23]. This precision allows for the detection of deglycosylation events (∼2.2 kDa shifts), oxidation (+16 Da), and aggregation states (dimers and trimers). Coupled with reverse-phase liquid chromatography (RP-LC), ESI-TOF-MS resolves heavy/light chains and glycoforms while preserving structural integrity, which is critical for biosimilars [24].

**Figure 4:**
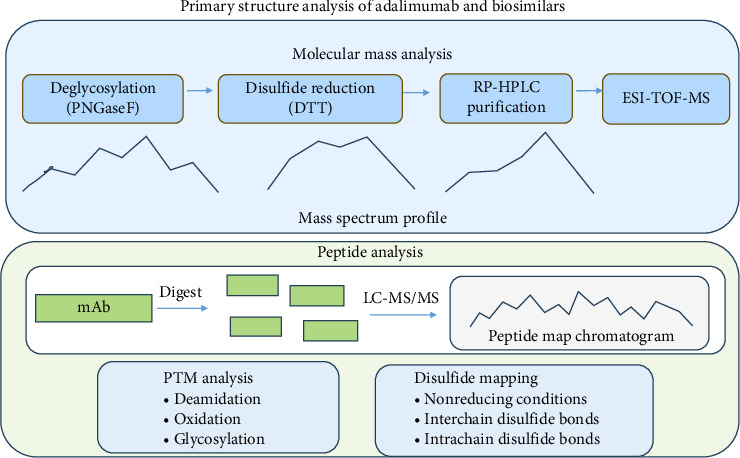
Process outline for the primary structure analysis of adalimumab and its biosimilars.

This technique has been critical in comparing the molecular mass profiles of Humira and its biosimilars, and the significant steps are discussed in the following (Figure 4). The initial step is the deglycosylation of the proteins using an enzyme, PNGaseF, to remove attached glycan moieties from the protein. This helps in generating a streamlined profile for comparison. This is followed by the disulfide reduction process using dithiothreitol (DTT) or tris(2-carboxyethyl)phosphine (TCEP), which destabilizes the disulfide bond that holds the antibody structure [25]. This separates the antibody into its constituent heavy chains (HCs) (∼50 kDa each) and light chains (LCs) (∼23 kDa each). Using reverse-phase high-performance liquid chromatography (RP-HPLC), the individual components are separated based on their hydrophobic interactions with a column, leading to a purified sample. ESI-TOF-MS analysis with Q-TOF or Orbitrap instruments was employed to determine molecular masses [24]. Isoelectric focusing is used to separate proteins based on their electrical charge across different pH levels (from 3 to 10), generating a “fingerprint” of the protein's charge distribution [25].

**Figure 5:**
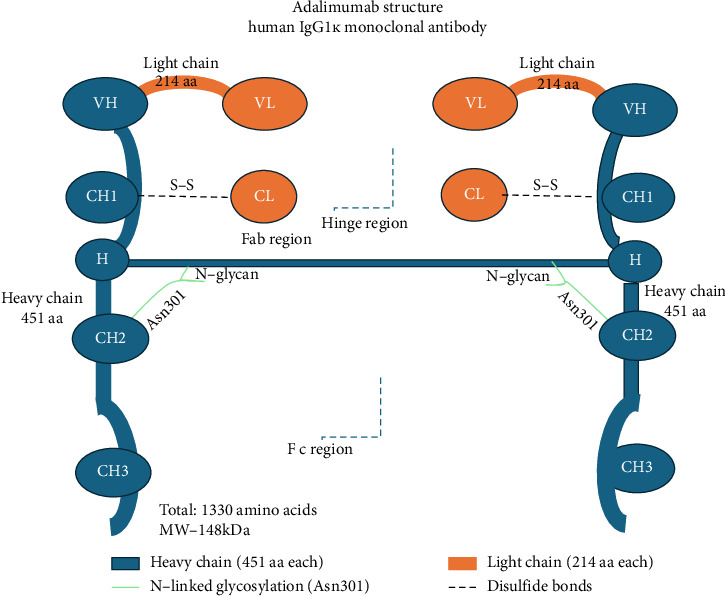
Molecular structure of adalimumab (Humira).

Humira is a human IgG1*κ* monoclonal antibody with a molecular weight of approximately ∼148 kDa with amino acid residues of 1330 (2 HCs of 451 amino acids each and two LCs of 214 amino acids each) (Figure 5). The variable regions of Humira and its biosimilars (VH and VL) and the complementarity-determining regions (CDRs), which are crucial for antigen binding, were similar in biosimilars. The constant regions of the heavy and LCs were identical, ensuring the antibodies have the same effector functions [25, 26]. Structural analysis of all four biosimilars demonstrated highly comparable mass values, with differences in the sub-Dalton range (1-2 Da in chains of ∼23,000–50,000 Da) (Table 2). A marginally higher intact mass was observed in MSB11022, and ABP501 demonstrated excellent similarity. The observed differences are minimal and fall within the expected analytical variability for monoclonal antibodies.

#### 2.1.2. Peptide Analysis

Peptide mapping provides an in-depth characterization of amino acid sequences and PTMs, essential for confirming protein identity and detecting potential impurities or modifications that may occur during manufacturing, formulation, or storage. Manufacturers employ sophisticated analytical techniques for Humira biosimilars (ABP501, FKB327, MSB11022, and SB5). In this process, the antibodies are broken down into smaller peptide fragments using enzymes like trypsin or pepsin, and these fragments are subsequently analyzed using techniques such as liquid chromatography coupled with tandem mass spectrometry, reverse-phase ultrahigh-performance liquid chromatography with tandem mass spectrometry (RP-UHPLC-MS/MS) and ion trap electrospray mass spectrometry [30].

These techniques allow for precise identification of the amino acid sequence of each fragment, PTMs such as deamidation (asparagine to aspartic acid conversion), oxidation (methionine residues), and glycosylation patterns at the Fc region (sugar structures). The disulfide mapping is also conducted under reducing and nonreducing conditions to confirm the presence or absence of disulfide bonds (critical for the three-dimensional structure) in the exact locations [31]. Even minor differences in these characteristics could potentially impact the activity and stability of the drug, which will, in turn, trigger varied immune responses in the host, altering the safety profile.

Humira has 142 identified peptides, with N-linked glycosylation at Asn301 in the CH2 domain of each HC [32]. Detailed peptide mapping results have demonstrated that all biosimilars (ABP501, FKB327, MSB11022, and SB5) maintained sequence identity with Humira, with minimal differences in PTM profiles (Table 2). These findings provide strong evidence for structural similarity at the molecular level, which is a critical component of the totality of evidence required for biosimilar approval by regulatory agencies.

### 2.2. HOS

Higher-order secondary and tertiary structures are critical for establishing biosimilarity between reference biologics and biosimilar counterparts. Various spectrophotometric techniques are employed to analyze these comparative structures of Humira with its biosimilars, ABP501, FKB327, MSB11022, and SB5.

#### 2.2.1. Secondary Structure Analysis

Fourier transform infrared spectroscopy (FTIR) is a powerful technique for determining secondary structure in proteins by measuring vibrational frequencies following chemical bonds' absorption of distinct wavelengths. When infrared radiation interacts with protein molecules, different structural elements (α-helices, β-sheets, turns, and random coils) produce characteristic absorption patterns in the amide I region (1700 − 1600 cm^−1^), creating a unique structural fingerprint [33]. For comparative analysis, Humira and biosimilars equivalent concentrations (typically 10 mg/mL) in their respective formulation buffers were measured using a Bruker Vertex 70 FTIR spectrometer equipped with a mercury–cadmium–telluride (MCT) detector. The spectral range scanned included 4000 − 850 cm^−1^, resolution of 4 cm^−1^ with 100 scans coadded per sample at a temperature of 25°C. A 9-point Savitzky–Golay smoothing algorithm was applied to enhance spectral quality without compromising structural information [27]. Spectral similarity was quantified using second-derivative analysis and correlation coefficient calculations using ThermoOMNIC software. The second-derivative spectra are particularly useful for enhancing subtle differences in peak positions and resolving overlapping bands [33].

Far UV Circular Dichroism (CD) spectroscopy measures secondary similarities in Humira biosimilars. This technique measures differential absorption (260-170 nm) of left and right circularly polarized light by peptide bonds in proteins. Different secondary structural elements generate characteristic spectral patterns, such as *α*-helices, which produce negative bands at 222 nm and 208 nm and a positive band at 193 nm. In comparison, β-sheets show a negative band at 218 nm and a positive band at 195 nm, and random coils display a negative band around 195 nm [34]. For analysis, Humira and biosimilars were diluted to 0.2–0.3 mg/mL in buffer solutions (low-concentration phosphate buffer without chloride ions) and measured using a Chirascan spectropolarimeter with a wavelength range of 260-190 nm, bandwidth of 1 nm, path length of 0.1 cm, and scan speed of 20 nm/min at a temperature of 25°C. CD spectra were normalized for concentration and path length and expressed as mean residue ellipticity [*θ*]_MRW_. Secondary structure content was estimated using deconvolution algorithms (CDNN, SELCON3, or CDSSTR) and compared.

The secondary structural analysis of FKB327 to Humira was carried out by FTIR and CD spectroscopy [28]. CD spectroscopy was used for the secondary structural analysis of ABP 501, MSB11022, and SB5 [25, 29, 35]. The detailed protein structural analysis, the identification of *α*-helices and β-strands, evaluation of dynamic stability, assessment of cofactor interactions, and investigation of folding patterns to be similar in Humira and biosimilars (Table 3). The FTIR spectral analysis revealed that all biosimilars exhibited strong correlation coefficients (> 0.99) with Humira, indicating highly similar secondary structure profiles. The dominant secondary structure element in all products was β-sheet, consistent with the expected structure of IgG antibodies. Minor variations in peak positions (within 2 cm^−1^) were within the accepted analytical variability and did not signify structural differences. The far-UV CD spectral analysis further confirmed the predominance of β-sheet structure in both Humira and its biosimilars. All biosimilars demonstrated comparable secondary structure distributions to Humira, with root-mean-square deviation (RMSD) values below 0.03, indicating high similarity. The characteristic negative band at ∼217 nm and the positive band near 195 nm were conserved across all products, confirming the preservation of the immunoglobulin (IgG) fold.

#### 2.2.2. Tertiary Structure Analysis

Near-UV CD and intrinsic fluorescence spectroscopy and hydrogen/deuterium exchange mass spectrometry were used for the tertiary structure analysis of Humira and its biosimilars [17, 25, 28, 29, 35]. Near-UV CD spectroscopy (320-250 nm) probes the aromatic amino acids for the presence of amino acids such as tryptophan (Trp) (290–305 nm), Tyr (275–285 nm), and Phe (255–270 nm) and disulfide bonds [36]. Samples of Humira and biosimilars (0.7 mg/mL) were analyzed using a Chirascan spectropolarimeter with a wavelength range of 320-250 nm. For analysis by intrinsic fluorescence spectroscopy, the natural fluorescence of aromatic amino acids (Trp) is used to probe the tertiary structure and conformational changes. The emission spectrum of Trp is highly sensitive to its environment and changes in the polarity or solvent exposure due to structural alterations shift the emission maximum [37]. During the analysis process, Humira and biosimilars (0.1 mg/mL) were diluted with deuterium oxide-based buffer to minimize interference, and fluorescence spectra were recorded using a spectrofluorometer at an excitation wavelength of 280 nm (for both Trp and Tyr) or 295 nm (Trp-selective) and emission range of 290–450 nm [25, 28, 29, 35]. Spectral parameters, including emission maximum wavelength (λmax), spectral shape, and fluorescence intensity, were compared between Humira and biosimilars. The comparative analysis of Humira with the biosimilars for tertiary structure analysis is given in Table 2.

Tertiary structure analysis with hydrogen/deuterium exchange mass spectrometry (HDX-MS) measures the rate of hydrogen atom exchange with deuterium atom in the protein backbone [29]. The factors influencing the exchange rate involve the hydrogen bonding, solvent accessibility, and structural dynamics. Well-structured regions with strong hydrogen bonding show slower exchange rates than flexible or solvent-exposed regions [38]. The Waters HDX-MS system, coupled to Synapt G2-Si mass spectrometer, was used for analysis of Humira and SB5. Samples were diluted to 1 mg/mL in phosphate buffer (pH 6.8). The HDX reaction was initiated by diluting the protein sample 1:20 in deuterated buffer, followed by incubation at 20°C for varying time points (10 s, 1 min, 10 min, 1 h, and 4 h). The reaction was quenched by reducing the pH to 2.5 and the temperature to 0°C. Proteins were then digested with pepsin, and LC-MS analyzed the resulting peptides. DynamX software was used to process HDX-MS data, calculating deuterium uptake, generating butterfly plots, and difference plots. Coverage maps were generated to verify adequate peptide coverage (91.1% of the HC sequence and 100% of the LC sequences). Difference plots highlighting regions with significant deuterium uptake differences (> 0.5 Da) were used to identify potential structural differences between Humira and SB5.

The tertiary structure analysis by near-UV CD spectral analysis demonstrated high similarity between Humira and all biosimilars, with similarity scores above 0.97 (Table 2). The spectral features confirmed that the amino acids Trp, tyrosine, and phenylalanine were largely conserved, indicating comparable aromatic amino acid environments. FKB327 showed minor variations in the phenylalanine region but remained within the acceptable range of similarity [28]. The intrinsic fluorescence analysis revealed highly similar emission maxima (*λ*_max_) for Humira and all biosimilars, with less than 0.5 nm differences. This indicates comparable microenvironments around Trp residues, suggesting conserved tertiary structures (Table 2). The relative fluorescence intensities and spectral moments were also statistically indistinguishable (*p* > 0.05), further supporting structural similarity. The HDX-MS analysis also confirmed the conformational dynamics and solvent accessibility of SB5 compared with Humira [29]. All regions showed differences in deuterium uptake below the significance threshold of 0.5 Da. The hinge region showed the highest variability, consistent with its inherent flexibility in IgG antibodies.

A comprehensive assessment of the HOS similarity between Humira and its biosimilars is summarized in Table 4. All four adalimumab biosimilars (ABP501, FKB327, MSB11022, and SB5) exhibited HOSs similar to the reference product Humira. Integrating multiple orthogonal spectroscopic techniques provided robust evidence for structural similarity at both secondary and tertiary levels.

### 2.3. PTMs

Most eukaryotic proteins undergo covalent modification after their ribosomal synthesis, known as PTMs. To date, several hundred PTMs have been identified, and these modifications consistently impact the structural and functional characteristics of the proteins. Specific enzymes are responsible for adding PTMs to proteins, predominantly focusing on the phosphorylation and ADP ribozylation of intracellular proteins and glycosylation, formation of disulfide linkages, and carboxylation of extracellular proteins. Therapeutic proteins often undergo several PTMs, such as carboxylation, hydroxylation, amidation, sulfation, disulfide bond formation, proteolytic processing, and glycosylation. Glycosylation is the most common and complex PTM, especially in biopharmaceuticals.

Chromatographic techniques such as RP-HPLC, hydrophobic interaction chromatography, and boronate affinity chromatography, combined with UV/fluorescence detection (FLD), are effective for quantifying oxidized and reduced species. When integrated with mass spectrometry, these methods enable precise identification, quantification, and separation of various product-related variants, such as oxidized, deaminated, isomerized, and glycated species, along with proteolytic fragments and misfolded proteins [39].

#### 2.3.1. Glycan Analysis

Glycan analysis, the study of glycans or oligosaccharides covalently bonded to proteins, is an essential component of biochemistry and biopharmaceuticals. Given that most proteins undergo some degree of glycosylation during synthesis, glycans fulfill various structural and functional roles in membrane and secreted proteins. They impact numerous biological and physiological processes, including cellular communication, growth and development, cellular immunity, recognition and regulatory activities, and gene expression. Monoclonal antibodies usually feature a single N-glycosylation site on each HC's Fc region, with the LCs typically unglycosylated. However, confirmation of additional glycosylation site(s) in the HCs is crucial [40]. Characterizing glycan structures, with particular attention to the levels of mannosylation, galactosylation, fucosylation, and sialylation, is essential. The significance of analyzing the distribution of glycan structures, majorly G0, G1, and G2, is emphasized by EMA/CHMP/BWP/532517/2008 Committee for Medicinal Products for Human Use [41]. Understanding these patterns will help determine the safety and efficacy of the biological behavior of monoclonal antibodies.

Glycan variation detection involves a multi-step analytical workflow that begins with the release of N-linked glycans from the protein backbone by PNGase F. This enzyme cleaves the bond between the asparagine residue and the innermost N-acetylglucosamine (GlcNAc). Once released, glycans undergo derivatisation by 2-aminobenzamide (2-AB) or procainamide, where a fluorophore is introduced for sensitive glycans detection [42].

Hydrophilic interaction liquid chromatography-high-performance liquid chromatography (HILIC-HPLC) detection technique was employed to analyze N-linked glycans in ABP501 [27]. This technique separates glycans based on their hydrophilicity, with more branched and sialylated structures retained longer on the liquid chromatography column. N-glycans are then detected using FLD for quantitative analysis and coupled with mass spectrometry (MS) for structural characterization through fragment ion analysis.

Reverse-phase HPLC was used to compare FKB327 with Humira , where the derivatized glycans interact with the hydrophobic stationary phase, and the separation is influenced by the hydrophobicity of the fluorescent label and the glycan structure [27, 28]. The analysis of glycans in SB5 was carried out by a unique technique of UPLC MS/MS and LC-ESI-MS/MS, which revealed the presence of two types of core fucosylated complex N-linked glycan at Asn297 and Asn301 in the HC region [29]. In MS-based techniques, mass-to-charge measurements are compared against glycan databases, which will allow the identification of specific structures. For site-specific glycan analysis, glycopeptides are analyzed after proteolytic digestion to analyze localized glycosylation sites [43].

Integrating these orthogonal methods provided a comprehensive profile of glycosylation patterns, allowing the detection of subtle variations in fucozylation, galactozylation, and sialylation between Humira and its biosimilars. The comparative analysis reveals that all four biosimilars demonstrate highly similar glycosylation profiles to Humira (Table 4). The key glycan structures in Humira G0F, G1F, and G2F forms had consistent distributions across all biosimilars. Minor variations were detected in some quantitative aspects, but these differences were within acceptable ranges that would not affect clinical efficacy or safety.

### 2.4. Product-Related Impurities

To ensure the quality of the biosimilars, the EMA has specified the necessity of analyzing impurities that arise during production, handling, and storage. These impurities affect the product's efficacy, safety, and stability [44]. Variants are assessed based on the differences in characteristics such as size (aggregates, fragments, and particulate matter) and charge (acidic and basic form). These variations lead to reduction, oxidation, glycation, and protein misfolding, causing minor biosimilars' chemical structure and composition changes. Various analytical methods, such as chromatography, MS, and spectrometry, are used to ensure the limits of purities during biosimilar manufacturing [45].

#### 2.4.1. Size Variants

Protein unfolding can form aggregation and fragments due to the varying environmental changes during manufacturing. These misfoldings trigger the immune response in patients. Aggregates are formed due to the shear, thermal, or chemical stress during the freeze-thaw cycles and can vary from soluble to visible forms. Size exclusion high-performance liquid chromatography (SE-HPLC) was used for size-based separation of molecules to compare the biosimilars of Humira along with ABP501, FKB327, MSB11022, and SB5.

Liu et al. [27] used SE-HPLC with light scattering to analyze ABP501 on an Agilent 1100 system, utilizing a Tosch Bioscience TSK-GEL G3000S WXL column for assessing size variants, including high molecular weight and monomer species. Capillary electrophoresis with sodium dodecyl sulfate (CE-SDS) was used to study protein loss and salt-induced aggregation during SE-HPLC. CE-SDS measures the number of protein fragments, nonglycosylated proteins, and partially reduced proteins. Under nonreducing conditions (nrCE-SDS), the purity of denatured intact antibodies and under reducing conditions (rCE-SDS), the intact LC, HC, and nonglycosylated HC (NGHC) were studied. The similarity of FKB327 to Humira was determined by Schreiber et al. [28] through CE and SE-HPLC based on molecular movement driven by an electromotive force in a polyacrylamide gel matrix within a fused silica capillary. Proteins were labeled with fluorescence under both conditions (nonreducing and reducing) and detected via laser-induced fluorescence with excitation at 488 nm and emission at 560 nm. SEC was used to analyze the size heterogeneity and purity of FKB327 and MSB11022 [25, 28]. Impurities were minimal, making up less than 1.7% of the areas in nonreducing conditions. In the analysis of SB5, the low molecular weights (%LMWs), %IgG, and %2HIL were measured using nrCE-SDS, and the main component (%LC+%HC) was assessed through rCE-SDS for SB5, all falling within predefined similarity ranges. SB5 exhibited minor differences in NGHC levels, which were insignificant as demonstrated in Table 5 [29].

Schreiber et al. [28] demonstrated the assessment of the size heterogeneity of FKB327 and adalimumab through field-flow fractionation (FFF), serving as an orthogonal method to SE-HPLC. The FFF analysis utilized a phosphate-buffered saline (PBS) mobile phase and UV absorbance monitoring at 215 nm for detection. FFF is a technique employed for submicron particle analysis, utilizing differential retention of biomolecules and particles due to external fields or gradients, such as gravitational, centrifugal, electrical, flow, and thermal forces. This chromatographic-like elution technique, devoid of solid phases, enables the analysis of biomolecules (e.g., proteins, peptides, and polysaccharides) and particles (e.g., latex, microbial, and parasites), typically ranging from 1 nm to 300 μm [28].

In 2017, Magnenat et al. [25] used AUC to demonstrate similar levels of monomers, aggregates, dimers, and fragments in MSB11022 and Humira. AUC stands out for its ability to analyze molecular size distribution directly, using absorbance, interference, and fluorescence optical systems within an ultracentrifuge for precise, real-time observations. It employs sedimentation velocity experiments, utilizing hydrodynamic theory to assess macromolecules' size, shape, and interactions based on their behavior in high centrifugal fields. Sedimentation equilibrium concentration gradient determination is a method that uses a lower centrifugal force to determine the molecular mass and solution characteristics. Size variants for SB5 were determined by SEC-coupled multiangle laser light scattering and sedimentation velocity analytical ultracentrifugation (SV-AUC). These techniques confirmed the similarity of the biosimilars with the reference product (Table 5).

#### 2.4.2. Charge Variants

Charge variant proteoforms are generated during biosimilar manufacturing in colloidal environments like buffers or formulations and are commonly analyzed by cation exchange chromatography (CEX). In CEX chromatography, the separation is based on differences in protein surface charge through ionic interactions with the negatively charged stationary phase [46]. The differential charge variants of ABP501 were compared with the original by cation exchange high-performance liquid chromatography (CEX-HPLC) on the ProPac WCX- 100 salt gradient column [27]. The purity of charge variants was quantified based on the peak area percentages. CEX-HPLC with DionexProPacWCX column, with NaCl gradient in sodium phosphate buffer (pH 6.5), was used, and absorbance peaks detected at 280 nm were used for analyzing the charge variants of SB5 [29]. Imaged capillary isoelectric focusing (icIEF) was also used to confirm the presence of variants in SB5. In icIEF, proteins are separated based on their isoelectric points (pI) in a pH gradient formed by ampholytes under an electric field [47]. Increased acidic and fewer basic variants were detected by this technique when compared to Humira (Table 6). Ten different charge variants were reported for MSB11022 after icIEF analysis. The pI ranged from 7.94 to 9.14 of these variants, and this matched those of Humira variants and was comparable to them. Capillary zone electrophoresis (CZE) is another process used to confirm the separation of proteins based on their charge-to-size ratio in free solution. In this technique, the samples (Humira and FKB327) were injected into the capillaries containing 0.1 M NaOH, water, and 400 mM *ε*-aminocaproic acid and 2 mM triethylenetetramine in 0.2% HPMC, pH 5.7–6.0 (Background electrolyte). The electrophoresis peaks were analyzed to determine the relative percentages of charge variants [28].

Analysis of these charge variants reveals that all four biosimilars demonstrate highly comparable charge distribution profiles to the reference product Humira (Table 6). ABP501 exhibited a charge variant profile nearly identical to the reference product, with only slightly reduced basic variants, likely due to differences in C-terminal lysine processing. FKB327 demonstrated comparable overall charge distribution, with acceptable variations in C-terminal lysine content. MSB11022 matched the pI of Humira with minor differences in deamidation-related acidic variants. SB5 showed a slightly higher proportion of the central peak (+2–3%) than the reference product, but the overall acidic and basic variant distributions remained within the similarity range. The analytical methods revealed that deamidation of asparagine residues and C-terminal lysine variability were the primary contributors to charge heterogeneity across all products, the consistent findings across multiple orthogonal analyses.

All platforms provide strong evidence for the similarity between Humira and its biosimilars in charge variants, supporting their overall comparability assessment. While minor differences in specific charge variants were observed, these variations were deemed within acceptable ranges that would not significantly impact clinical performance, as confirmed by subsequent functional and clinical studies for all four biosimilars.

### 2.5. Process-Related Variants

Process-related variants or residuals include components such as HCPs, HCD, cell culture materials, and downstream processing remnants. These arise from cell culture and downstream processing. In biosimilarity studies, sensitive and efficient methods such as enzyme-linked immunosorbent assay (ELISA) and quantitative PCR are commonly used to assess these. However, due to the complexity of HCP mixtures, qualitative techniques are being supplemented with proteomic methods, such as 2D gel electrophoresis and LC-MS/MS. Recent studies have utilized ELISA, 2D-PAGE/DIGE, and LC-MS/2D-LCMSE to identify and quantify these variants [27] effectively.

The safety and efficacy of biopharmaceuticals can be significantly affected by HCPs, as even smaller residue concentrations affect biologics' stability, leading to toxicity and immunogenicity. Therefore, it is vital to identify and quantify HCPs during production to ensure product quality. HCP analysis is the key to quality control to confirm that levels remain within acceptable limits and meet regulatory standards.

Particles and aggregates linked to ABP 501 and adalimumab reference products were examined through various methods to detect and quantify particles and aggregates of spherical and nonspherical shapes and sizes. The subvisible particles were assessed by light obscuration and microflow imaging (MFI), and submicron particles by dynamic light scattering (DLS). The presence of small-sized silicon oil droplets or protein-origin nonspherical particles was determined after analysis [27]. The quantitative measurement of ABP501 was done by HCP-ELISA using a polyclonal anti-HCP profile, and inverted two-dimensional liquid chromatography (2D-LC) with online MS employing data-independent acquisition (MSE) was used for detection and quantification of HCPs. The 2D-DIGE was carried out to confirm the absence of HCP in formulations. Residual DNA content was measured by polymerase chain reaction. To analyze process-related impurities during FKB327, a commercial ELISA kit, and residual DNA content were measured by threshold assay [28]. Compendial light obscuration assay was used for analyzing the presence of subvisible particles of sizes ≥ 2 μm, ≥ 5 μm, ≥ 10 μm, and ≥ 25 μm and MFI was used for confirming the particles of size less than ≥ 5 μm [28]. The absorbance at 280 nm was used for analyzing the protein concentration, and the degradation and stability profile was confirmed by assessing them under accelerated (25°C) and stressed (40°C) conditions.

## 3. Biological Activity

Binding assays determine the binding kinetics of the products to their specific receptors during preclinical studies. The binding behaviors of TNF *α* inhibitors are analyzed by two significant in vitro biological assays: the receptor-binding assay and the cell apoptosis assay. The receptor-binding assay provides a quantitative evaluation of the binding strength of the TNF *α* receptor. In contrast, the cell apoptosis assay offers a qualitative assessment of the functional strength by evaluating the potential of these inhibitors to trigger apoptosis in TNF *α*-expressing cells. Combined, these assays help understand the therapeutic potential for treating conditions associated with excessive TNF *α* activity.

### 3.1. Binding Kinetics

Their FcRn pathway activity analyses the pharmacokinetics and therapeutic effectiveness of TNF *α* inhibitors. The binding kinetics to the neonatal Fc receptor (FcRn) is crucial, as it directly impacts the serum half-life of antibodies, their therapeutic dosing intervals, and the overall drug exposure. A stronger binding to FcRn leads to a longer circulation time for the antibodies, which can reduce the frequency of dosing for patients [48]. The relative binding assays for ABP501 were conducted by quantifying the binding affinity of soluble TNF *α* to the FcRn [49]. The recombinant IgG was tagged with fluorescent and expressed in 293T cell lines. Notably, 293T cells, a modified variant of human embryonic kidney cells, have been explicitly engineered to display FcRn on their surface for such analyses [50]. The determination of relative binding was facilitated using ELISA methodology, ensuring a robust and quantifiable measurement of interaction between the recombinant IgG and the targeted receptors. In addition, for FKB327, surface plasmon resonance (SPR) analysis was carried out to determine the binding affinity of human Fc gamma receptor (FcγR) and FcRn [28]. His-tagged human Fcγ receptors and FcRn were added to a BIAcore TM biosensor chip immobilized with anti-FCy His antibody. The equilibrium dissociation constant (KD) was calculated using specific binding models for FcγRs and FcRn.

To assess Fab binding potency for MSB11022, cytotoxicity was measured using L929-A9 cells. The assessment included measuring affinity to TNF, Fcg RI, Fcg RIIa, Fcg RIIb, Fcg RIIIa, Fcg RIIIb, and neonatal FcR. The assessment of interactions with these Fcγ receptors yields critical information on the key immunological processes, including antibody-dependent cellular cytotoxicity (ADCC), complement-dependent cytotoxicity (CDC), and other immune-mediated effector functions. Understanding these interactions is crucial for assessing the therapeutic efficacy and safety profiles of biosimilars, as they play a significant role in the overall performance and reliability of these biologic agents [51]. The affinity was measured using SPR [25]. The binding of FcγRIIa, FcγRIIb, and FcγRIIIa was measured using an AlphaScreen-based binding assay. In this, the fluorescent signal obtained after the interaction with human IgG1 mAb-coated acceptor beads and reduced glutathione-coated donor beads was used for measurement, and this signal inversely correlates with Fc receptor binding activity. The quantitative measure of binding activity was calculated relative to the reference standard using parallel line analysis (PLA) software. The binding activity of SB5 was examined using an FcRn-binding assay and measured using AlphaScreen-based assays. The binding potency to FcrγRIa was determined by cell-based luciferase reporter assay with a specific cell line. The soluble TNF *α* binding activity was analyzed using solid phase ELISA and measured using SPR analysis, fluorescent activated cell sorting, and fluorescent resonance energy transfer (FRET)-based competitive inhibition assay. The activity of SB5 on transmembrane TNF *α* was determined through flow cytometry, and its binding affinity to FcγRIIIa or FcγRIIIb was measured by SPR [29]. C1q binding activity was also determined by the layered ELISA method. These diverse approaches comprehensively determined the interaction of these biological agents with their target molecules.

The integration of various complementary techniques, including ELISA, SPR, AlphaScreen, FRET, and flow cytometry, facilitated a comprehensive assessment of the binding profiles of the analyzed biosimilars. This multifaceted approach enhances the reliability of the findings, as each method possesses distinct strengths and limitations that contribute uniquely to the analysis.

SPR is widely recognized as the gold standard for binding kinetics analysis due to its ability to provide real-time, label-free measurements of both association and dissociation rates. The BIAcore platform employed for FKB327 is particularly well established within regulatory environments, aligning with the recommendations from the FDA and EMA guidelines for biosimilar characterization. This methodology allows for the precise determination of dissociation constant (KD) values by previous studies [52, 53].

AlphaScreen assays complement this by offering high sensitivity for detecting protein–protein interactions, making them especially suitable for high-throughput screening of binding affinities. The observed inverse correlation between the signal and Fc receptor binding activity for MSB11022 aligns with the anticipated mechanisms of this platform, and AlphaScreen has been demonstrated in numerous studies to effectively characterize therapeutic antibodies [54].

In addition, the utilization of engineered 293T cells expressing FcRn for ABP501 and L929-A9 cells for assessing cytotoxicity related to MSB11022 introduces functional relevance that extends beyond mere binding affinity measurements. These cell-based methods have proven effective in validating the characterization of TNF-α inhibitors [55]. Furthermore, the cell-based luciferase reporter assay conducted for SB5's binding to FcγRIa provides a functional readout that correlates with downstream signaling processes, rather than just binding events. Collectively, these analyses bolster the assertion of similarity between these biosimilars and their reference products.

### 3.2. In Vitro Potency

The potency of ABP 501 was examined by apoptosis inhibition assay in the human histiocytic lymphoma cell line U-937 [27]. Evaluations included ADCC and CDC using Chinese hamster ovary (CHO) target cells, specifically the CHO M7 variant (by Amgen), known for expressing an uncleaved, membrane-bound form of TNFα. The test sample's cytotoxicity was calculated and expressed as a percentage of relative cytotoxicity. FKB327's effector function activity was assessed through a cell-based assay involving the tmTNF-α EL4 cell line, and apoptosis induction was quantitatively analyzed using flow cytometry [28]. The CDC activity of FKB327 was explored to determine the similarities to Humira. At the same time, the neutralization efficiency against rhtnf-mediated cytotoxicity was examined by adding varying concentrations of rhTNF-α solution (0.06–1000 ng/mL) to the L929 cell line in the presence of actinomycin D. The subsequent assessment of cell viability was conducted by the quantification of intracellular adenosine triphosphate (ATP) levels.

The biological activity of MSB11022 was analyzed in the murine fibroblast L929-A9 cell line based on its ability to inhibit cytotoxicity in the presence of a fixed TNF-cycloheximide concentration [25]. To analyze the inhibitory activity of SB5 on the soluble TNF-α signaling pathway, a TNF-α neutralization assay was carried out in the 293-NF-κB-luc cell line, with the TNF-α neutralization potency determined using the Steady-Glo Luciferase Assay System from Promega [29]. Furthermore, regulatory macrophages were induced from human PBMC using adalimumab samples in a mixed lymphocyte reaction (MLR), and their numbers were measured by flow cytometry, calculating the sample's relative activity using mean fluorescence intensity of a specific marker against the reference standard. The antiproliferative effect of adalimumab-treated induced regulatory macrophage T cells was observed through bidirectional MLR. For apoptosis activity measurement, Caspase 3/7 activity in a colon cancer cell line was assessed using adalimumab samples, with the inhibitory effect calculated relative to the reference standard via PLA software. IL-8 release inhibition was measured in an in vitro IBD model utilizing a specific cell line treated with TNF-α and adalimumab samples, with the production of IL-8 measured via an ELISA kit and relative activity determined based on optical density (OD) compared with the reference standard. To assess TNF-α-induced adhesion molecule expression inhibition, soluble VCAM-1 expression levels in endothelial cells were measured, determining sample activity based on OD values compared with the reference. In vitro potencies analyzed by these methodologies further confirmed the functionality of these biosimilars with their reference products.

## 4. In Vivo (Animal) Studies

Animal, pharmacologic, and toxicokinetic data are crucial to obtaining the nod for clinical trials. Studies on different animal species provide a brief idea of how the molecule will perform in the clinical trial and give the investigators insight regarding the human equivalent dose of the test article. However, the main drawback of animal studies of biosimilars is the interspecies variation in receptor morphology and pharmacokinetics; thus, newer guidelines are wavering on animal data requirements.

### 4.1. Repeat-Dose Toxicity Study

Toxicokinetic evaluation was performed for ABP 501 as part of a repeat dose toxicity program in cynomolgus monkeys [56]. While evaluating the toxicological studies, Amgen, Inc. gave monkeys 157 mg/kg of ABP 501, adalimumab, or a vehicle via subcutaneous injection for 1 month, like the highest dose used in the Humira development program. No significant differences were found in TK parameters between animals dosed with ABP 501 and adalimumab (US). Local tolerance at the SC injection site was assessed through histology in a 1-month toxicology study on monkeys. Observations of focal fibroplasia/fibrosis and focal mononuclear or mixed cell infiltrates at the SC injection site were attributed to the injection process itself, as these effects are typical at injection sites and were similarly seen in both the vehicle control and the ABP 501 and adalimumab (USA) groups. Despite the different formulations of ABP 501 and adalimumab (USA), no significant difference in local tolerance between the two drugs was noted.

To understand the toxicity of FKB327, a 4-week comparative repeat-dose toxicity study was conducted by Mylan GmbH in macaques to confirm the similarity of FKB327 to the original Humira for biosimilar use [57]. The study included comparisons of toxicokinetics, local tolerance, and potential immunotoxic profiles. The cynomolgus monkey was chosen, and a dose of 30 mg/kg was administered, representing a 12-fold margin of safety when converted to the equivalent human dose of 40 mg. The safety margins for 80 and 160 mg doses were 6 and 3, respectively. The weekly dose was double that of patients, and the intended route of administration for FKB327 in humans was chosen. FKB327 and US Humira were well tolerated at 30 mg/kg doses. Various evaluations found no significant treatment-related effects or differences between FKB327 and Humira. Some histopathological changes were observed, but they were considered due to exaggerated pharmacological effects rather than toxicity. Antidrug antibodies were evaluated, showing minimal relevance in humans. Genotoxicity, carcinogenicity, and reproductive toxicity have not been studied.

In a repeated dose toxicity study, three male and three female macaque groups received weekly subcutaneous (SC) injections of either vehicle control MSB11022 (32 mg/kg) or Humira-USA (32 mg/kg), a total of five injections. The manufacturer provided toxicokinetic data collected in the study, showing similar pharmacokinetic behavior for both products. The results indicate a comparable exposure, immunogenicity, and toxicity profile for Idacio and Humira-US, with no adverse events [58].

The pharmacokinetic program for SB5 included a comparison of the toxicokinetic profiles between SB5 and US Humira in macaques within a GLP-compliant 4-week repeat-dose toxicity study [58]. While pharmacokinetic studies are not required for biosimilars in the EU, toxicokinetic analysis was conducted. This was part of the efforts to develop SB5 for the global market. The absence of studies on the distribution, metabolism, excretion, and pharmacokinetic interactions aligns with the Committee for Human Medicines guidelines. A 4-week comparative GLP-compliant repeat-dose toxicity study in macaques confirmed the similarity between SB5 and the original Humira (US origin) for biosimilar application. For SB5, the study compared the toxicokinetics, local tolerability, and potential immunotoxic profiles of SB5 and US-Humira. Both were well tolerated at a 32 mg/kg dose (subcutaneously, once for 4 weeks), aligning with previous adalimumab studies in cynomolgus monkeys, with no unexpected outcomes. There were no significant or biologically relevant treatment-related effects or differences in clinical findings, body weight, food consumption, ophthalmoscopy, electrocardiography, hematology, coagulation, clinical chemistry, urinalysis endpoints, peripheral blood leukocyte count, or macroanalysis between SB5 and US-Humira. Observed minor random changes were attributed to biological interindividual variability or were considered random or procedural, not treatment related. Histopathological examination of the injection sites showed similar types and frequencies of findings in animals treated with SB5 and Humira.

### 4.2. Toxicokinetic Studies

In the in vivo study, no significant differences were observed between FKB327 and US Humira at 1 and 10 mg/kg doses. Statistical analysis showed no significance between FKB327 and EU-Humira at either dose level. No further discussion was deemed necessary by the CHMP.

The PK and TK data for FKB327 were obtained from a study on human TNF-α transgenic mice, a single-dose PK study in cynomolgus monkeys, and a TK comparison of FKB327 and US Humira in cynomolgus monkeys during a 4-week repeat-dose toxicity study [59]. Though PK studies are not explicitly required for biosimilar development, TK analysis was conducted for FKB327's global registration. The studies utilized validated ECL methods to detect FKB327 and US Humira and antibodies against these in mouse and nonhuman primate sera. Validation studies also backed the assay's use for adalimumab and its antibody quantification. The PK/TK comparisons showed no significant differences between FKB327 and Humira. However, these in vivo studies involved a limited animal sample, so claiming ‘bioequivalence' between FKB327 and Humira is impossible. The document suggests referring to human bioequivalence studies instead. The lack of studies on distribution, metabolism, excretion, and PK drug interactions aligns with CHMP guidelines for similar biological medicinal products containing monoclonal antibodies [59].

In a study using the Tg197 mouse model established for assessing the impact of immunomodulatory agents on arthritis, the efficacy of MSB11022 and Humira was evaluated [60]. The analysis focused on their effects on body weight, arthritis severity, and histopathological scores. Results revealed the dose-dependent effectiveness of both compounds in these areas, with statistical analysis showing similarity in outcomes for body weight, overall arthritis score, and total histopathology score at week 12. In addition, in the Tg197/TNBS colitis model, both substances influenced body weight variations and TNFα release in colonic organ culture, yet this effect did not follow a dose-response pattern.

The in vivo efficacy study for SB5 was conducted in the Tg197 transgenic mouse model of arthritis, which closely resembles human RA pathology due to overexpression of human TNF-α [61]. SB5 and US Humira were administered intraperitoneally at doses of 0.5 mg/kg, 3 mg/kg, and 10 mg/kg twice weekly before the onset of arthritic symptoms to assess their protective effect. The treatment lasted 7 weeks, with treatment responses evaluated weekly from ages 3–10 weeks through macroscopic arthritis scores and at the end of treatment through histopathological scores from posterior ankle joints, all done in a blinded assessment. The study found that the in vivo efficacy of SB5 in preventing arthritis is similar to that of US Humira compared with control mice receiving a vehicle.

## 5. Discussion

Biosimilar development offers an affordable alternative to expensive biologicals while ensuring quality in the pharmaceutical industry. The complexity of the biologicals presents significant challenges to the development of biosimilars. Unlike generics, they must mimic the originals in safety, purity, and potency. In the preclinical evaluation, the regulatory safety and efficacy standards must also be met, apart from establishing the similarity. Increased market acceptance of biosimilars has created a need for a more transparent regulatory framework. Due to the biosimilar interchangeability, peer-reviewed research is essential for understanding global regulations. These studies provide a comprehensive understanding of the implementation of advanced analytical tools for research and development, indicating the favorable adoption of new technologies. A comprehensive framework is established by integrating applied and proposed methodologies in biosimilarity assessments. An extensive literature search unveils various analytical techniques and biological assays used in biosimilar analysis. The evolution of the similarity assessment platform focuses on utilizing mixed methods, combining separation techniques with mass spectrometric detection. This cost-effective and userfriendly approach is widely embraced for its versatility and broad applicability. Furthermore, advancements in biopharmaceutical analysis through technologies such as 2D NMR, multidimensional chromatography and HDX-MS have enriched our understanding of CQAS. These techniques aim to facilitate cost-effective comparisons for biosimilars by reducing the reliance on extensive tools, thus enhancing data quality.

Humira represents the inaugural human-based therapeutic intervention to achieve a significant clinical remission rate. This therapeutic agent's efficacy and safety profile have been well established through rigorous clinical trials, thus making it a favored biological disease-modifying antirheumatic drug (DMARD). In the wake of its success, several biosimilars have been developed for this TNFα inhibitor, setting a new standard for subsequent therapeutic interventions. These biosimilars, ABP501, FKB327, MSB11022, and SB5, have demonstrated similar biological responses across several investigations. These studies have shown similar biological responses across a series of investigations. These studies have encompassed proliferative activity, receptor-binding affinity toward TPO-R and FcRn, and phosphorylation processes involving TPO-R and JAK2. The comparative analysis showcases that these biosimilars exhibit parallel biological activity in both in vitro assays and preclinical studies. The latter were conducted on rats and cynomolgus monkeys, with findings indicating that both categories of the therapeutics instigated dose-dependent augmentation in platelet proliferation without manifesting notable differences. Moreover, the pharmacokinetic and pharmacodynamic profiles of these biosimilars, as evaluated in cynomolgus monkeys, were congruent, thereby suggesting equivalent in vitro activities and in vivo efficacy profiles compared with Humira in animal models.

This review article is designed to serve as an invaluable asset for the biopharmaceutical industry, supplying critical insights for researchers, academicians, regulatory bodies, and manufacturing entities engaged in developing or enhancing the analytical platforms for assessing biosimilars. This compendium facilitates informed decision-making regarding investing in analytical tools and adopting uniform methodologies. Furthermore, it contributes to an augmented comprehension of the biosimilar domain, emphasizing the increasing significance of sophisticated technologies for comprehensive analysis.

## Figures and Tables

**Table 1:** Overview of biosimilars developed for Humira.

	Humira biosimilars	Manufacturer	US approval date	EU approval date
1	ABRILADA (adalimumab-afzb)	PFIZER	2019	2020
2	AMJEVITA (adalimumab-atto)—ABP501	AMGEN	2016	2017
3	HADLIMA (adalimumab-bwwd)	SAMSUNG BIOEPIS	2019	2017
4	IDACIO (adalimumab-aacf)-MSB11022	FRESENIUS KABI	2022	2019
5	HULIO (adalimumab-fkjp)-FKB327	MYLAN	2020	2018
6	HYRIMOZ (adalimumab-adaz)	SANDOZ	2018	2023
7	YUFLYMA (adalimumab-aaty)	CELLTRION	2023	2021
8	YUSIMRY (adalimumab-aqvh)	COHERUS	2021	2023
9	CYTELZO (adalimumab-adbm)	Boehringer ingelheim pharmaceuticals, inc.	2017	2017
10	SIMLANDI (adalimumab-ryvk)	Alvotech/Teva	2024	2024

**Table 2:** Comparative analysis of the primary structure of Humira and its biosimilars.

Intact mass analysis					
Biosimilars	Measured intact mass (kDa)	Mass deviation from reference		Primary glycoforms	Reference
Humira (reference)	148.0 ± 0.2	—		G0F, G1F, G2F	[25, 27–29]
ABP501	148.1 ± 0.3	+0.1 kDa		G0F, G1F, G2F	[27]
FKB327	147.9 ± 0.2	−0.1 kDa		G0F, G1F	[28]
MSB11022	148.2 ± 0.3	+0.2 kDa		G0F, G1F, G2F	[25]
SB5	148.0 ± 0.2	0.0 kDa		G0F, G1F, G2F	[29]

**Subunit analysis (reduced conditions)**					
**Biosimilars**		**Heavy chain (kDa)**		**Light chain (kDa)**	**HC/LC ratio**

Humira (reference)		50.6 ± 0.1		23.4 ± 0.1	2.16
ABP501		50.7 ± 0.1		23.4 ± 0.1	2.17
FKB327		50.5 ± 0.2		23.3 ± 0.1	2.17
MSB11022		50.8 ± 0.2		23.5 ± 0.1	2.16
SB5		50.6 ± 0.1		23.4 ± 0.1	2.16

**Peptide mapping and mass fingerprinting**					
**Biosimilars**	**Sequence coverage**	**Number of identified peptides**		**Posttranslational modifications**	

Humira (reference)	98.5%	142		N-glycosylation, C-terminal lysine variants, oxidation (M, W), deamidation (N, Q)	
ABP501	99.0%	145		Similar to reference with slightly lower deamidation	
FKB327	98.2%	140		Similar to reference with slightly higher oxidation sites	
MSB11022	98.7%	143		Similar to reference with comparable PTM profile	
SB5	98.8%	144		Similar to reference with slightly lower C-terminal lysine variants	

**Glycosylation profile analysis**					
**Biosimilars**	**G0F (%)**	**G1F (%)**	**G2F (%)**	**Man5 (%)**	**Other (%)**

Humira (reference)	42 ± 3	41 ± 3	12 ± 2	1.5 ± 0.5	3.5 ± 1.0
ABP501	45 ± 3	39 ± 3	11 ± 2	1.8 ± 0.6	3.2 ± 1.0
FKB327	48 ± 3	40 ± 3	7 ± 2	2.0 ± 0.5	3.0 ± 1.0
MSB11022	40 ± 3	43 ± 3	13 ± 2	1.2 ± 0.5	2.8 ± 1.0
SB5	43 ± 3	40 ± 3	12 ± 2	1.6 ± 0.5	3.4 ± 1.0

**Table 3:** Comparative secondary and tertiary analysis of Humira and its biosimilars.

**Second derivative FTIR spectra of Humira and biosimilars in the amide I region**					
**Biosimilars**	**Peak positions (cm ** ^**−**^ **^1^)**	**Secondary structure assignment**		**Correlation coefficient vs. humira**	

Humira (reference)	1639, 1687	β-sheet (dominant)		1.000	
ABP501	1638, 1686	β-sheet (dominant)		0.998	
FKB327	1640, 1687	β-sheet (dominant)		0.997	
MSB11022	1639, 1688	β-sheet (dominant)		0.996	
SB5	1637, 1685	β-sheet (dominant)		0.995	

**Far-UV CD spectra of Humira and biosimilars**					
**Biosimilars**	**α-helix (%)**	**β-sheet (%)**	**Turn (%)**	**Random coil (%)**	**RMSD vs. humira**

Humira (reference)	9.3 ± 0.5	64.2 ± 1.1	12.8 ± 0.7	13.7 ± 0.8	—
ABP501	9.1 ± 0.6	65.0 ± 1.3	12.5 ± 0.9	13.4 ± 0.7	0.015
FKB327	9.5 ± 0.4	63.8 ± 1.2	13.0 ± 0.8	13.7 ± 0.9	0.018
MSB11022	9.0 ± 0.7	64.5 ± 1.0	12.6 ± 0.6	13.9 ± 0.8	0.021
SB5	9.4 ± 0.5	64.0 ± 1.4	12.9 ± 0.7	13.7 ± 0.6	0.014

**Near-UV CD spectra of Humira and biosimilars**					
**Biosimilars**	**Tryptophan region (290–305 nm)**	**Tyrosine region (275–285 nm)**	**Phenylalanine region (255–270 nm)**	**Similarity score vs. humira**	

Humira (reference)	Characteristic negative peak	Double negative peaks	Multiple fine structures	1.000	
ABP501	Conserved	Conserved	Conserved	0.989	
FKB327	Conserved	Conserved	Minor variations	0.973	
MSB11022	Conserved	Conserved	Conserved	0.985	
SB5	Conserved	Conserved	Conserved	0.981	

**Intrinsic fluorescence emission spectra of Humira and biosimilars**					
**Biosimilars**	***λ***_max_ **(nm)**	**Relative intensity (%)**	**Spectral moment**	**Statistical similarity (** **p** **-value)**	

Humira (reference)	336.5 ± 0.3	100	Reference	—	
ABP501	336.7 ± 0.2	98.7 ± 1.8	0.998	> 0.05 (NS)	
FKB327	337.0 ± 0.4	97.5 ± 2.1	0.991	> 0.05 (NS)	
MSB11022	336.3 ± 0.3	101.2 ± 1.9	0.996	> 0.05 (NS)	
SB5	336.8 ± 0.2	99.3 ± 1.5	0.997	> 0.05 (NS)	

**Differential HDX-MS analysis between Humira and SB5**					
**Region**	**Peptides analyzed**	**Average difference in deuterium uptake (Da)**		**Statistical significance**	

VH domain (1–120)	28	0.11 ± 0.09		NS	
CH1 domain (121–220)	23	0.14 ± 0.10		NS	
Hinge region (221–240)	5	0.22 ± 0.15		NS	
CH2 domain (241–350)	32	0.18 ± 0.14		NS	
CH3 domain (351–451)	39	0.09 ± 0.08		NS	
VL domain (1–110)	27	0.13 ± 0.11		NS	
CL domain (111–214)	35	0.10 ± 0.09		NS	

**Table 4:** Comprehensive analysis of higher-order structure and glycan characterization for Humira and its biosimilars.

Comprehensive assessment of higher-order structure similarity between Humira and biosimilars				
Biosimilar	Secondary structure similarity		Tertiary structure similarity	Overall HOS similarity
ABP501	High (> 99%)		High (> 98%)	Highly similar
FKB327	High (> 99%)		High (> 97%)	Highly similar
MSB11022	High (> 99%)		High (> 98%)	Highly similar
SB5	High (> 99%)		High (> 98%)	Highly similar

**Comparative analysis of glycan characterization methods for Humira and its biosimilars**				
**Biosimilar**	**Primary analytical methods**	**Key glycan attributes analyzed**	**Method specificity/sensitivity**	**Distinguishing methodological features**

Humira (reference)	RP-HPLC-MS, HILIC-UPLC, LC-ESI-MS	G0, G1, G2 distribution, core fucosylation, afucosylation, high mannose	High resolution with dual detection capabilities	Established as reference standard methodology
ABP501	HILIC-HPLC, MS, NP-HPLC with fluorescence detection	N-linked glycans at Asn297, G0F, G1F, G2F ratios, high mannose content	Enhanced sensitivity for minor glycan species detection	Combined orthogonal approaches for comprehensive glycan mapping
FKB327	RP-HPLC, CE-SDS, SEC-MALS	Core fucosylation, terminal galactosylation, mannose content	Moderate sensitivity with high reproducibility	Emphasized chromatographic separation over mass spectrometric identification
SB5	UPLC-MS/MS, LC-ESI-MS/MS	Core fucosylated complex N-linked glycans at Asn297 and Asn301, G0F, G1F, G2F distribution	High resolution with dual confirmation capabilities	Unique focus on site-specific glycosylation at both Asn297 and Asn301
MSB11022	MS-based glycoprofiling, HILIC-UPLC	G0F, G1F, G2F distribution, molecular mass deviation (+0.2 kDa)	High precision (148.2 ± 0.3 kDa mass determination)	Integration of accurate mass determination with glycan profiling for enhanced structural characterization

**Table 5:** Comparative analysis of size variant characterization of Humira and its biosimilars.

Comparative analysis of size variant characterization of Humira and its biosimilars					
Biosimilar	Primary analytical methods	Secondary/orthogonal methods	Size variants detected	Key findings	Technical specifications
Humira (reference)	SE-HPLC, CE-SDS (nonreducing and reducing)	AUC, SEC-MALS	High molecular weight species (HMWS), monomers, fragments	Established baseline for comparison	Various columns and methods across studies
ABP501	SE-HPLC with light scattering	CE-SDS (nonreducing and reducing)	HMWS, monomers, fragments, nonglycosylated proteins	Comparable monomer content and aggregate profiles to reference	Agilent 1100 system with TSK-GEL G3000S WXL column
FKB327	CE-SDS, SE-HPLC	Field-flow fractionation (FFF)	Intact antibodies, LC, HC, aggregates	Impurities < 1.7% under nonreducing conditions; comparable size distribution to reference	CE with fluorescence labeling (ex: 488 nm, em: 560 nm); FFF with PBS mobile phase and UV detection at 215 nm
MSB11022	SEC, AUC	Dynamic light scattering (DLS), atomic force microscopy (AFM)	Monomers, aggregates, dimers, fragments, subvisible particles	Similar levels of monomers (∼98–99%), aggregates (< 2%), and fragments; comparable hydrodynamic radius	AUC with sedimentation velocity experiments; DLS for particle size distribution in solution; AFM for surface morphology characterization
SB5	nrCE-SDS, rCE-SDS, SEC-MALS	SV-AUC	%LMW, %IgG, %2HIL, %LC, %HC, NGHC	Minor differences in NGHC levels (not significant); other parameters within predefined similarity ranges	SEC coupled with multiangle laser light scattering; sedimentation velocity AUC

**Table 6:** Comparative analysis of charge variant analysis of humira and its biosimilars.

Charge variant analysis of Humira and its biosimilars				
Biosimilar	Primary analytical methods	Charge variants detected	Key findings	Technical specifications
Humira (reference)	CEX-HPLC, icIEF, CZE	Acidic variants, main peak, basic variants	Established baseline: ∼25–30% acidic, ∼55–60% main, ∼10–15% basic variants	Various platforms with pH gradient 7–10; salt gradient for CEX
ABP501	CEX-HPLC, icIEF	Acidic variants (deamidated/sialylated), main peak, basic variants (C-terminal lysine)	Highly similar charge distribution to reference; slightly lower basic variants (∼1–2% difference)	CEX with ProPac WCX-10 column; icIEF with pI markers 7.0–10.0
FKB327	CZE, icIEF	Acidic, main, and basic charge variants	Comparable charge variant profile; differences in C-terminal lysine content within acceptable range	CZE with bare fused silica capillary; icIEF with fluorescent pI markers
MSB11022	CEX-HPLC, imaged cIEF	Acidic variants, main peak, basic variants	Matching isoelectric point (pI); comparable acidic/basic variant distribution with minor differences in deamidation	CEX with salt gradient elution; cIEF with whole column imaging capability
SB5	icIEF, CEX-HPLC	Acidic, main, and basic charge variants	Within similarity range for acidic and basic variants; slightly higher main peak proportion (+2–3%)	icIEF with pH 3–10 ampholytes; CEX with pH gradient elution

## Data Availability

Data sharing is not applicable to this article as no datasets were generated or analyzed during the current study.

## References

[1] Cessak G., Kuzawińska O., Burda A. (2014). TNF Inhibitors–Mechanisms of Action, Approved and Off-Label Indications. *Pharmacological Reports*.

[2] Kleef R., Hager E. D. (2006). Fever, Pyrogens and Cancer. *Medical Intelligence Unit*.

[3] Miesfeld R., Okret S., Wikström A. C., Wrange O., Gustafsson J.-Å., Yamamoto K. R. (1984). Characterization of a Steroid Hormone Receptor Gene and mRNA in Wild-type and Mutant Cells. *Nature*.

[4] Wang X., Lin Y. (2008). Tumour Necrosis Factor and Cancer, Buddies or Foes? 1. *Acta Pharmacologica Sinica*.

[5] Zhao Y., Zhang T., Shen X. (2022). Tumor Necrosis Factor Alpha Delivers Exogenous Inflammation-Related microRNAs to Recipient Cells with Functional Targeting Capabilities. *Molecular Therapy*.

[6] Jiang Y., Yu M., Hu X. (2017). STAT1 Mediates Transmembrane TNF-Alpha-Induced Formation of Death-Inducing Signaling Complex and Apoptotic Signaling via TNFR1. *Cell Death & Differentiation*.

[7] Jang D. I., Lee A. H., Shin H. Y. (2021). The Role of Tumor Necrosis Factor Alpha (TNF-α) in Autoimmune Disease and Current TNF-α Inhibitors in Therapeutics. *International Journal of Molecular Sciences*.

[8] Goyal A., Cusick A. S., Thielemier B. (2023). *ACE Inhibitors*.

[9] Zhang H., Shi N., Diao Z., Chen Y., Zhang Y. (2021). Therapeutic Potential of TNFα Inhibitors in Chronic Inflammatory Disorders: Past and Future. *Genes & diseases*.

[10] Lopetuso L. R., Cuomo C., Mignini I., Gasbarrini A., Papa A. (2023). Focus on Anti-tumour Necrosis Factor (TNF)-α-related Autoimmune Diseases. *International Journal of Molecular Sciences*.

[11] Neri P., Zucchi M., Allegri P., Lettieri M., Mariotti C., Giovannini A. (2011). Adalimumab (Humira™): a Promising Monoclonal Anti-tumor Necrosis Factor Alpha in Ophthalmology. *International Ophthalmology*.

[12] Gibbons J. B. (2023). Humira: The First $20 Billion Drug. *American Journal of Managed Care*.

[13] Bandyopadhyay A. (2013). Complexities of Biosimilar Product. *Journal of Bioanalysis & Biomedicine*.

[14] European Medicines Agency (2015). Annual Activity Report 2015. https://www.ema.europa.eu/en/about-us/annual-reports-work-programmes.

[15] Food and Drug Administration (2015). Scientific Considerations in Demonstrating Biosimilarity to a Reference Product. *Guidance for Industry*.

[16] Central Drugs Standard Control Organization CDSCO and Department of Biotechnology DBT (2016). Guidelines on Similar Biologics: Regulatory Requirements for Marketing Authorization in India.

[17] Nupur N., Joshi S., Gulliarme D., Rathore A. S. (2022). Analytical Similarity Assessment of Biosimilars: Global Regulatory Landscape, Recent Studies and Major Advancements in Orthogonal Platforms. *Frontiers in Bioengineering and Biotechnology*.

[18] Us Department of Health and Human Services (2001). Bioanalytical Method Validation, Guidance for Industry.

[19] Alberts B., Johnson A., Lewis J., Raff M., Roberts K., Walter P. (2002). Analyzing Protein Structure and Function. *Molecular Biology of the Cell*.

[20] D’Atri V., Imiołek M., Quinn C. (2024). Size Exclusion Chromatography of Biopharmaceutical Products: From Current Practices for Proteins to Emerging Trends for Viral Vectors, Nucleic Acids and Lipid Nanoparticles. *Journal of Chromatography A*.

[21] Mehta A., Silva L. P. (2015). MALDI-TOF MS Profiling Approach: How Much Can We Get from it?. *Frontiers of Plant Science*.

[22] Den Engelsman J., Garidel P., Smulders R. (2011). Strategies for the Assessment of Protein Aggregates in Pharmaceutical Biotech Product Development. *Pharmaceutical Research*.

[23] Ho C. S., Lam C. W. K., Chan M. H. (2003). Electrospray Ionisation Mass Spectrometry: Principles and Clinical Applications. *Clinical Biochemist Reviews*.

[24] Nupur N., Chhabra N., Dash R., Rathore A. S. (2018). Assessment of Structural and Functional Similarity of Biosimilar Products: Rituximab as a Case Study. *mAbs*.

[25] Magnenat L., Palmese A., Fremaux C. (2017). Demonstration of Physicochemical and Functional Similarity between the Proposed Biosimilar Adalimumab MSB11022 and Humira. *mAbs*.

[26] Valido A., Araújo F. C., Eurico Fonseca J., Gonçalves J. (2019). A Review of Adalimumab Biosimilars for the Treatment of Immune-Mediated Rheumatic Conditions. *EMJ Rheumatology*.

[27] Liu J., Eris T., Li C., Cao S., Kuhns S. (2016). Assessing Analytical Similarity of Proposed Amgen Biosimilar ABP 501 to Adalimumab. *BioDrugs*.

[28] Schreiber S., Yamamoto K., Muniz R., Iwura T. (2020). Physicochemical Analysis and Biological Characterization of FKB327 as a Biosimilar to Adalimumab. *Pharmacology research & perspectives*.

[29] Lee N., Lee J.Ah J., Yang H. (2019). Evaluation of Similar Quality Attribute Characteristics in SB5 and Reference Product of Adalimumab. *mAbs*.

[30] Neagu A. N., Jayathirtha M., Baxter E., Donnelly M., Petre B. A., Darie C. C. (2022). Applications of Tandem Mass Spectrometry (MS/MS) in Protein Analysis for Biomedical Research. *Molecules (Basel)*.

[31] Tsai P. L., Chen S. F., Huang S. Y. (2013). Mass Spectrometry-Based Strategies for Protein Disulfide Bond Identification. *Reviews in Analytical Chemistry*.

[32] U.S. Food and Drug Administration/Center for Drug Evaluation and Research (2019). Product Quality Review(s): Application Number 761059 Orig1s000.

[33] Rieppo L., Saarakkala S., Närhi T., Helminen H. J., Jurvelin J. S., Rieppo J. (2012). Application of Second Derivative Spectroscopy for Increasing Molecular Specificity of Fourier Transform Infrared Spectroscopic Imaging of Articular Cartilage. *Osteoarthritis and Cartilage*.

[34] Zsila F. (2022). Far-UV Circular Dichroism Signatures Indicate Fluorophore Labeling Induced Conformational Changes of Penetratin. *Amino Acids*.

[35] Doshi S., Wang H., Chow V. (2022). Establishing PK Equivalence between Adalimumab and ABP 501 in the Presence of Antidrug Antibodies Using Population PK Modeling. *Clinical Therapeutics*.

[36] Kelly S. M., Price N. C. (2000). The Use of Circular Dichroism in the Investigation of Protein Structure and Function. *Current Protein & Peptide Science*.

[37] Munishkina L. A., Fink A. L. (2007). Fluorescence as a Method to Reveal Structures and Membrane-Interactions of Amyloidogenic Proteins. *Biochimica et Biophysica Acta, Biomembranes*.

[38] Wei H., Mo J., Tao L. (2014). Hydrogen/deuterium Exchange Mass Spectrometry for Probing Higher Order Structure of Protein Therapeutics: Methodology and Applications. *Drug Discovery Today*.

[39] Arndt J. R., Wormwood Moser K. L., Van Aken G. (2021). High-resolution Ion-Mobility-Enabled Peptide Mapping for High-Throughput Critical Quality Attribute Monitoring. *Journal of the American Society for Mass Spectrometry*.

[40] Boune S., Hu P., Epstein A. L., Khawli L. A. (2020). Principles of N-Linked Glycosylation Variations of IgG-Based Therapeutics: Pharmacokinetic and Functional Considerations. *Antibodies*.

[41] Chmp T. T. (2004). *Committee for Medicinal Products for Human Use (CHMP)*.

[42] Fischler D. A., Orlando R. (2019). *N*-Linked Glycan Release Efficiency: A Quantitative Comparison between NaOCl and PNGase F Release Protocols. *Journal of Biomolecular Techniques: Journal of Biochemistry *.

[43] Liu G., Cheng K., Lo C. Y., Li J., Qu J., Neelamegham S. (2017). A Comprehensive, Open-Source Platform for Mass Spectrometry-Based Glycoproteomics Data Analysis. *Molecular & Cellular Proteomics*.

[44] Pokar D., Rajput N., Sengupta P. (2020). Industrial Approaches and Consideration of Clinical Relevance in Setting Impurity Level Specification for Drug Substances and Drug Products. *International Journal of Pharmaceutics*.

[45] Molineau J., Hideux M., West C. (2021). Chromatographic Analysis of Biomolecules with Pressurized Carbon Dioxide Mobile Phases–A Review. *Journal of Pharmaceutical and Biomedical Analysis*.

[46] Fekete S., Beck A., Veuthey J. L., Guillarme D. (2015). Ion-exchange Chromatography for the Characterization of Biopharmaceuticals. *Journal of Pharmaceutical and Biomedical Analysis*.

[47] Ghizzani V., Ascione A., Gonnella F., Massolini G., Luciani F. (2025). Exploring Imaged Capillary Isoelectric Focusing Parameters for Enhanced Charge Variants Quality Control. *Frontiers in Chemistry*.

[48] Andersen J. T., Dalhus B., Viuff D. (2014). Extending Serum Half-Life of Albumin by Engineering Neonatal Fc Receptor (FcRn) Binding. *Journal of Biological Chemistry*.

[49] Velayudhan J., Chen Y.-F., Rohrbach A. (2016). Demonstration of Functional Similarity of Proposed Biosimilar ABP 501 to Adalimumab. *BioDrugs*.

[50] Hubbard J. J., Pyzik M., Rath T. (2020). FcRn Is a Cd32a Coreceptor that Determines Susceptibility to Igg Immune Complex–Driven Autoimmunity. *Journal of Experimental Medicine*.

[51] Abdeldaim D. T., Schindowski K. (2023). Fc-engineered Therapeutic Antibodies: Recent Advances and Future Directions. *Pharmaceutics*.

[52] Sedger L. M., McDermott M. F. (2014). TNF and TNF-Receptors: From Mediators of Cell Death and Inflammation to Therapeutic Giants–Past, Present and Future. *Cytokine & Growth Factor Reviews*.

[53] Liu L., Stadheim A., Hamuro L. (2011). Pharmacokinetics of IgG1 Monoclonal Antibodies Produced in Humanized Pichia pastoris with Specific Glycoforms: a Comparative Study with CHO Produced Materials. *Biologicals*.

[54] Eglen R. M., Reisine T., Roby P. (2008). The Use of AlphaScreen Technology in HTS: Current Status. *Current Chemical Genomics*.

[55] Kaymakcalan Z., Sakorafas P., Bose S. (2009). Comparisons of Affinities, Avidities, and Complement Activation of Adalimumab, Infliximab, and Etanercept in Binding to Soluble and Membrane Tumor Necrosis Factor. *Clinical Immunology*.

[56] European Medicines agency CHMP Assessment Report AMGEVITA International Non-proprietary Name: Adalimumab Procedure No. EMEA/H/C/004212/0000. https://www.ema.europa.eu/en/documents/assessment-report/amgevita-epar-public-assessment-report_en.pdf.

[57] European Medicines agency CHMP Assessment Report Hulio International Non-Proprietary Name: Adalimumab. https://www.ema.europa.eu/en/documents/assessment-report/hulio-epar-public-assessment-report_en.pdf.

[58] European Medicines agency CHMP Assessment Report, Idacio International Non-Proprietary Name: Adalimumab.

[59] European Medicines agency CHMP Assessment Report, Imraldi International Non-Proprietary Name: Adalimumab. https://pdf4pro.com/amp/view/assessment-report-european-medicines-agency-1002ca.html.

[60] Puri A., Niewiarowski A., Arai Y. (2017). Pharmacokinetics, Safety, Tolerability and Immunogenicity of FKB327, a New Biosimilar Medicine of adalimumab/Humira, in Healthy Subjects. *British Journal of Clinical Pharmacology*.

[61] Ema/Chmp/Bmwp Guideline on Similar Biological Medicinal Products Containing Monoclonal Antibodies.

